# Dexmedetomidine modulates gut microbiota and improves long-term survival in sepsis patients with pre-existing malignancies: a propensity-matched analysis

**DOI:** 10.3389/fmicb.2025.1720867

**Published:** 2025-11-26

**Authors:** Yisen Zeng, Huiling Wu, Huaijian Zhang, Xiuhong Ye, Ling Chen, Yibin Ye

**Affiliations:** 1 Intensive Care Unit, Zhangzhou Affiliated Hospital of Fujian Medical University, Zhangzhou, Fujian, China; 2 Department of Anesthesiology, The First Affiliated Hospital of Xiamen University, School of Medicine, Xiamen University, Xiamen, Fujian, China; 3 Department of Anesthesiology, Zhangzhou Affiliated Hospital of Fujian Medical University, Zhangzhou, Fujian, China; 4 Department of Cardiac Surgery, Fujian Medical University Union Hospital, Fuzhou, Fujian, China

**Keywords:** tumor, sepsis, gut microbial colonization, dexmedetomidine, host-microbiota interaction mechanisms, clinical microbiota interventions, tumor-microbiota axis, microbiota-based therapy

## Abstract

**Background:**

The interplay between sedative agents and the gut microbiome may influence long-term outcomes in sepsis, but data are scarce. This study compared the effects of dexmedetomidine vs. propofol sedation on long-term survival in mechanically ventilated sepsis adults, with an exploratory focus on the gut microbiome and pre-existing malignancies.

**Methods:**

In this multicenter, retrospective cohort study, 1,295 mechanically ventilated adults with sepsis (2013–2020) were analyzed. Propensity score matching (1:1) balanced 27 baseline covariates, producing 177 matched pairs. Primary outcomes were 30-day, 90-day, and 5-year mortality. Secondary outcomes included delirium/coma-free days, cardiovascular safety, and 6-month functional status. Subgroup analyses assessed pre-existing malignancies and high antibiotic exposure (≥7 days before enrollment) as proxies for microbiome disruption. Gut microbiota composition was characterized via 16S rRNA sequencing in a pre-specified subcohort (*n* = 89).

**Results:**

After matching, dexmedetomidine was associated with significantly lower 5-year mortality (34.5% vs. 45.2%; HR 0.64, 95% CI 0.52–0.79; *p* = 0.039). Survival curves progressively diverged beyond 180 days. No differences were observed in short-term neurological outcomes or cardiovascular safety. Subgroup analyses showed enhanced survival benefits with dexmedetomidine in patients aged >65, females, those with pulmonary-source sepsis, SOFA >10, baseline delirium, pre-existing malignancies (OR 2.10, 95% CI 1.15–3.85; *p* = 0.015), and high antibiotic exposure as a proxy for gut dysbiosis (OR 1.95, 95% CI 1.08–3.52; *p* = 0.028). Exploratory 16S rRNA analysis in a subset (*n* = 89) revealed that dexmedetomidine was associated with enriched beneficial genera such as *Faecalibacterium* and *Bifidobacterium*, while propofol correlated with increased *Enterococcus*and *Escherichia/Shigella*.

**Conclusions:**

Dexmedetomidine sedation is associated with a significant 5-year survival benefit in mechanically ventilated sepsis patients, particularly among those with malignancies or factors predisposing to gut dysbiosis. The observed modulation of the gut microbiome toward a more symbiotic state provides a plausible mechanistic insight into these clinical findings, highlighting a potential role for microbiota-centric strategies in critical care.

## Introduction

Sepsis, a life-threatening dysregulated host response to infection, imposes substantial global mortality, particularly among mechanically ventilated patients requiring meticulous sedation management (Fleischmann et al., 2015; Dhital et al., 2018; Vincent et al., 2014). Current international guidelines strongly advocate light sedation using non-benzodiazepine agents—dexmedetomidine or propofol—to mitigate delirium risk and enhance ventilator synchrony in critical illness (Devlin et al., 2018). While both agents are first-line options, their comparative impact on sepsis-specific outcomes remains inadequately resolved. Mechanistically, dexmedetomidine's α_2_-adrenergic agonism confers unique immunomodulatory and neuroprotective properties, potentially attenuating sepsis-associated neuronal apoptosis and inflammatory cascades. Propofol, though pharmacokinetically favorable, lacks these targeted anti-inflammatory effects and carries metabolic risks (Kelbel et al., 1999; Memiş et al., 2007; Venn et al., 2001; Qiao et al., 2009; Nishina et al., 1999). Despite preclinical evidence suggesting dexmedetomidine's theoretical advantages, high-quality randomized trials (e.g., MENDS2, DESIRE) report equipoise in short-term mortality and delirium outcomes between these sedatives (Pandharipande et al., 2007; Riker et al., 2009; Jakob et al., 2012; Kawazoe et al., 2017; Shehabi et al., 2019). This inconsistency highlights critical knowledge gaps regarding long-term survival, functional recovery, and subgroup therapeutic benefits—an unresolved dilemma complicating evidence-based sedation strategies in sepsis.

The existing literature is notably constrained by methodological limitations. Prior trials prioritized 90-day endpoints, potentially overlooking delayed survival advantages; incorporated open-label designs and sedative crossovers; and inadequately addressed heterogeneity in sepsis prespecified subgroup. Crucially, no study has evaluated 5-year survival—a clinically meaningful endpoint reflecting chronic critical illness trajectories (Pandharipande et al., 2007; Riker et al., 2009; Jakob et al., 2012; Kawazoe et al., 2017). Furthermore, while subgroup analyses from SPICE III hinted at potential sepsis-specific benefits with dexmedetomidine, these lacked statistical power (Shehabi et al., 2019). Propensity-matched observational studies offer methodological advantages for balancing baseline confounders and examining long-term outcomes but remain scarce. Furthermore, emerging evidence underscores the gut microbiome as a pivotal modulator of sepsis outcomes, influencing systemic inflammation, immune response, and organ dysfunction. Antibiotic-induced dysbiosis, characterized by depletion of commensals like Bacteroides and Lactobacillus and enrichment of pathobionts such as Enterobacteriaceae, exacerbates sepsis severity and mortality. Similarly, malignancies alter host immunity and microbiome composition, potentially interacting with sedative choice to shape long-term survival. Dexmedetomidine's α_2_-adrenergic properties may mitigate gut barrier disruption and dysbiosis, whereas propofol's emulsion formulation might promote pathogenic overgrowth. However, no study has integrated microbial data into comparative sedation outcomes in sepsis.

To address these evidence gaps, we conducted a multicenter, propensity-matched cohort study of 1,295 mechanically ventilated sepsis adults. We hypothesized that dexmedetomidine would confer superior survival and neurological outcomes vs. propofol, attributable to its immunometabolic modulation. Primary objectives were threefold: (1) quantify differential effects on 30-day/90-day/5-year survival; (2) assess delirium/coma-free days and functional recovery; and (3) identify clinical prespecified subgroup deriving maximal benefit. By employing high-fidelity propensity matching across 27 covariates—including illness severity, prior sedative exposure, and delirium status—this study aims to overcome limitations of previous observational analyses and provide actionable insights for precision sedation in sepsis.

## Results

### Patient characteristics

**Figure 1:**
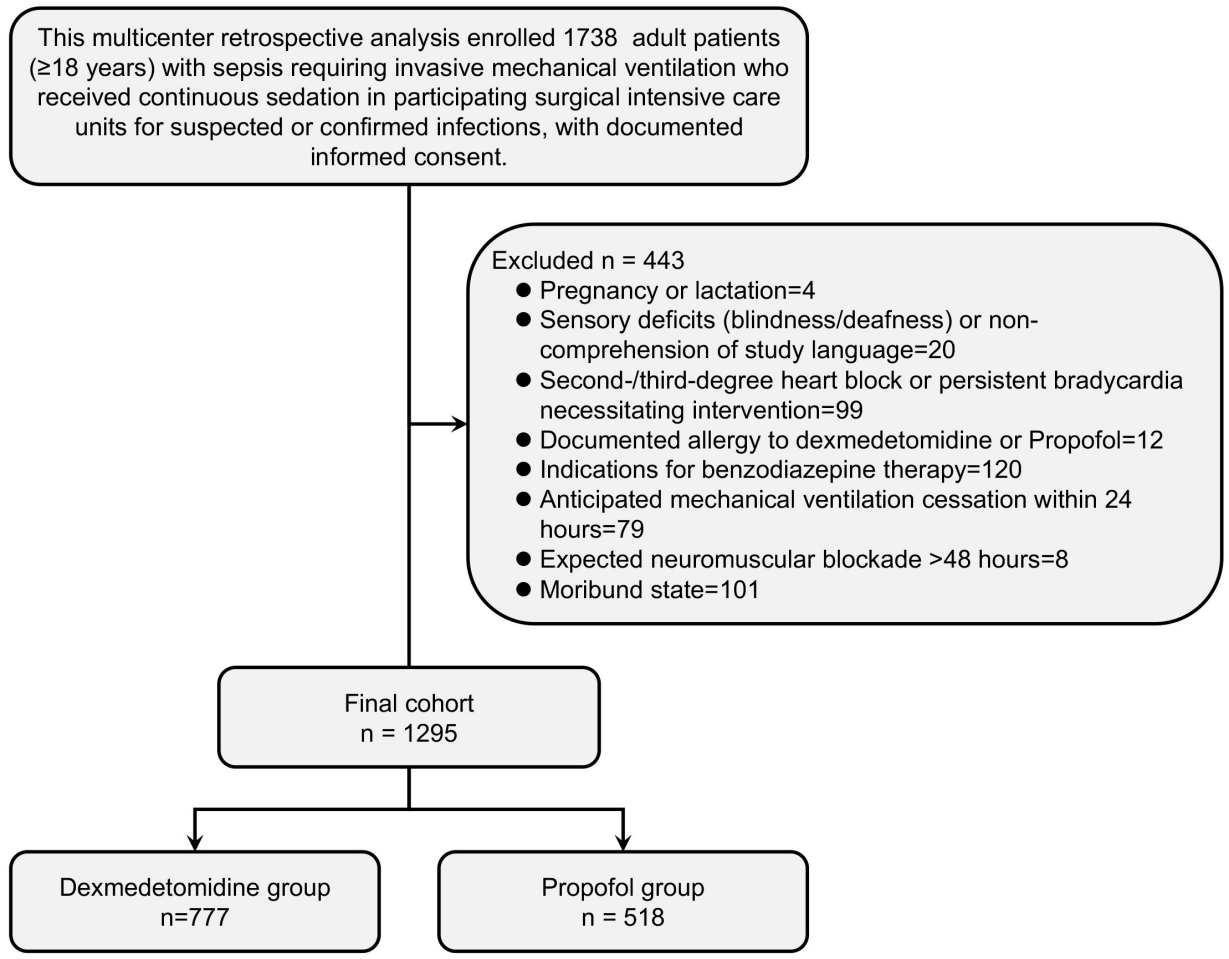
Patient Enrollment and Exclusion Flowchart. This CONSORT-compliant flowchart details patient selection for the multicenter retrospective analysis. Initially, 1,738 adults (≥18 years) with sepsis requiring invasive mechanical ventilation were screened across participating ICUs. All enrolled patients had documented informed consent and received continuous sedation for suspected or confirmed infections. After applying exclusion criteria, 443 patients (25.5%) were excluded for the following clinically significant reasons: indications for benzodiazepine therapy (*n* = 120, 27.1%), moribund state (*n* = 101, 22.8%), persistent bradycardia requiring intervention (*n* = 99, 22.3%), anticipated ventilation cessation ≤24 h (*n* = 79, 17.8%), sensory/language barriers (*n* = 20, 4.5%), documented sedation allergies (*n* = 12, 2.7%), pregnancy/lactation (*n* = 4, 0.9%), and expected prolonged neuromuscular blockade (*n* = 8, 1.8%). The final analytical cohort comprised 1,295 patients, stratified into dexmedetomidine (*n* = 777) and propofol (*n* = 518) treatment groups for subsequent propensity matching and outcome analyses.

As shown in Table 1 and Figure 1, prior to propensity matching, this multicenter cohort comprised 1,295 mechanically ventilated adults with sepsis (dexmedetomidine: *n* = 777; propofol: *n* = 518). Substantial baseline imbalances were observed between cohorts. Patients receiving dexmedetomidine demonstrated greater illness severity, evidenced by significantly higher APACHE II scores (27.90 ± 6.07 vs. 26.28 ± 5.83; *p* < 0.001), total SOFA scores (10.98 ± 3.19 vs. 9.94 ± 2.93; *p* < 0.001), and inflammatory markers including procalcitonin (2.72 ± 1.11 vs. 1.54 ± 0.28 μg/L; *p* < 0.001), CRP (152.26 ± 47.75 vs. 121.88 ± 41.05 mg/L; *p* < 0.001), and leukocyte counts (13.66 ± 4.82 vs. 11.81 ± 4.08 × 10^9^/L; *p* < 0.001). The dexmedetomidine cohort was older (62.89 ± 9.93 vs. 57.86 ± 9.65 years; *p* < 0.001) with higher prevalence of diabetes mellitus (44.4% vs. 32.1%; *p* < 0.001), coronary heart disease (40.0% vs. 28.8%; *p* < 0.001), and COPD (28.7% vs. 18.2%; *p* < 0.001). Notably, dexmedetomidine recipients exhibited lower baseline delirium prevalence (33.1% vs. 44.4%; *p* < 0.001) despite greater cardiorespiratory compromise, reflected in reduced PaO_2_/FiO_2_ ratios (179.18 ± 50.07 vs. 198.46 ± 46.80; *p* < 0.001). Prior medication exposures differed significantly, with dexmedetomidine patients more frequently receiving prior dexmedetomidine (17.4% vs. 9.1%; *p* < 0.001) but less frequently administered benzodiazepines (24.1% vs. 36.9%; *p* < 0.001). Paradoxically, despite greater baseline morbidity, the dexmedetomidine group demonstrated lower crude 90-day mortality (32.2% vs. 39.4%; *p* = 0.008) and overall mortality (36.7% vs. 47.3%; *p* < 0.001).

**Table 1:** The baseline clinical characteristics of patients.

**Subgroup**	**Dexmedetomidine (*n =* 777)**	**Propofol (*n =* 518)**	*P* value^*^
**Baseline characteristics**			
Age (years)	62.89 ± 9.93	57.86 ± 9.65	< 0.001
BMI, kg/m^2^	24.96 ± 5.49	24.90 ± 5.77	0.652
Sex			0.150
Female	327 (42.08)	239 (46.14)	
Male	450 (57.92)	279 (53.86)	
PaO2 FiO2 ratio	179.18 ± 50.07	198.46 ± 46.80	< 0.001
Procalcitonin	2.72 ± 1.11	1.54 ± 0.28	< 0.001
WBC	13.66 ± 4.82	11.81 ± 4.08	< 0.001
CRP	152.26 ± 47.75	121.88 ± 41.05	< 0.001
Diabetes mellitus	345 (44.40)	166 (32.05)	< 0.001
Hypertension	498 (64.09)	307 (59.27)	0.079
Coronary heart disease	311 (40.03)	149 (28.76)	< 0.001
COPD	223 (28.70)	94 (18.15)	< 0.001
Liver dysfunction	149 (19.18)	104 (20.08)	0.689
Renal dysfunction	162 (20.85)	125 (24.13)	0.164
IQCODE-SF score	3.07 ± 0.29	3.07 ± 0.31	0.965
Charlson comorbidity index	2.05 ± 1.17	1.96 ± 1.14	0.182
APACHE II score	27.90 ± 6.07	26.28 ± 5.83	< 0.001
Days from ICU admission to enrollment	1.33 ± 0.68	1.29 ± 0.72	0.321
Days of mechanical ventilation before enrollment	1.08 ± 0.54	1.03 ± 0.56	0.140
Total SOFA score at enrollment	10.98 ± 3.19	9.94 ± 2.93	< 0.001
Shock at enrollment, *n* (%)	439 (56.50)	299 (57.72)	0.663
Pre-existing malignancy, *n* (%)	89 (11.5%)	51 (9.8%)	0.342
High antibiotic exposure^*^, *n* (%)	312 (40.2%)	198 (38.2%)	0.491
**Infection characteristics**			
Source of infection, *n* (%)			0.638
Abdomen	43 (5.53)	25 (4.83)	
Blood	140 (18.02)	87 (16.80)	
Lung	320 (41.18)	204 (39.38)	
Other	60 (7.72)	52 (10.04)	
Skin_or_Wound	50 (6.44)	35 (6.76)	
Stool	33 (4.25)	17 (3.28)	
Urinary tract	131 (16.86)	98 (18.92)	
Infection status, *n* (%)			0.515
Confirmed by culture	495 (63.71)	329 (63.51)	
Ruled out	50 (6.44)	26 (5.02)	
Suspected but not confirmed	232 (29.86)	163 (31.47)	
**Prior medication exposure**			
Prior dexmedetomidine, *n* (%)	135 (17.37)	47 (9.07)	< 0.001
Prior propofol, *n* (%)	473 (60.88)	318 (61.39)	0.852
Prior benzodiazepine, *n* (%)	187 (24.07)	191 (36.87)	< 0.001
Prior opioid, *n* (%)	540 (69.50)	350 (67.57)	0.463
Prior antipsychotic, *n* (%)	91 (11.71)	73 (14.09)	0.207
Delirium at enrollment, *n* (%)	257 (33.08)	230 (44.40)	< 0.001
**Level of arousal at enrollment**			0.419
Agitated (RASS +1 to +4)	17 (2.19)	13 (2.51)	
Awake and calm (RASS 0)	49 (6.31)	36 (6.95)	
Coma (RASS −5 or −4)	262 (33.72)	191 (36.87)	
Deep sedation (RASS −3)	123 (15.83)	89 (17.18)	
Light sedation (RASS −2 or −1)	326 (41.96)	189 (36.49)	
**Treatment characteristics**			
Hours from inclusion to drug start, median	23.16 ± 9.05	22.79 ± 8.93	0.465
Days of drug administration, median	3.76 ± 1.67	3.93 ± 1.71	0.082
Daily dose, median	0.31 ± 0.12	10.91 ± 3.34	< 0.001
Number of drug adjustments, median	11.32 ± 6.63	11.52 ± 6.74	0.598
Drug temporarily held, *n* (%)	213 (27.41)	150 (28.96)	0.544
Drug permanently discontinued, *n* (%)	100 (12.87)	72 (13.90)	0.593
Median RASS score, median	−1.49 ± 1.02	−1.93 ± 1.01	< 0.001
Percent time at target sedation, median	60.11 ± 14.34	59.84 ± 13.89	0.731
Daily remifentanil dose, μg/hr, median	68.80 ± 29.54	57.01 ± 24.06	< 0.001
Midazolam used, *n* (%)	365 (46.98)	232 (44.79)	0.439
Antipsychotic used, *n* (%)	343 (44.14)	202 (39.00)	0.066
**Safety endpoints**			
Hypotension, *n* (%)	242 (31.15)	174 (33.59)	0.356
Bradycardia, *n* (%)	145 (18.66)	101 (19.50)	0.707
ARDS, *n* (%)	158 (20.33)	133 (25.68)	0.024
Hypertriglyceridemia, *n* (%)	65 (8.37)	77 (14.86)	< 0.001
Low cortisol, *n* (%)	185 (23.81)	90 (17.37)	0.006
**Outcomes**			
Days alive without delirium or coma (0–14 days), median	10.30 ± 2.39	9.13 ± 2.80	< 0.001
Ventilator-free days at 28 days, median	21.00 ± 4.38	19.42 ± 5.05	< 0.001
TICS-T score at 6 months, median	40.52 ± 11.65	41.58 ± 11.28	0.103
EQ-5D score at 6 months, median	0.64 ± 0.18	0.63 ± 0.18	0.230
Katz ADL score at 6 months, median	3.37 ± 1.24	3.45 ± 1.19	0.269
FAQ score at 6 months, median	12.33 ± 5.58	11.94 ± 5.50	0.219
**Death rate**			
In-hospital mortality	114 (14.67)	84 (16.22)	0.449
Death at 90 days, *n* (%)	250 (32.18)	204 (39.38)	0.008
Overall mortality	285 (36.68)	245 (47.30)	< 0.001

BMI, Body Mass Index; ADL, Activities of Daily Living; APACHE II, Acute Physiology and Chronic Health Evaluation II; ARDS, Acute Respiratory Distress Syndrome; CAM-ICU, Confusion Assessment Method for the ICU; CPOT, Critical-Care Pain Observation Tool; Dex, Dexmedetomidine; EQ-5D, European Quality of Life-5 Dimensions; FAQ, Functional Activities Questionnaire; ICU, Intensive Care Unit; IQCODE-SF, Informant Questionnaire on Cognitive Decline in the Elderly Short Form; Prop, Propofol; RASS, Richmond Agitation-Sedation Scale; SOFA, Sequential Organ Failure Assessment; TICS-T, Telephone Interview for Cognitive Status (total score).

^*^High antibiotic exposure defined as ≥7 days of systemic antibiotics prior to enrollment.

### Propensity-matched cohort characteristics

As shown in Table 2, following 1:1 propensity score matching (*n* = 177 per group), baseline characteristics were effectively balanced between dexmedetomidine and propofol recipients, with all standardized mean differences (SMD) below 0.10 post-matching. The cohorts demonstrated statistically comparable demographics, including age (60.98 ± 9.90 vs. 60.16 ± 9.10 years; SMD −0.090; *p* = 0.418), BMI (24.68 ± 5.39 vs. 24.58 ± 5.17 kg/m^2^; SMD −0.018; *p* = 0.870), and sex distribution (female: 47.5% vs. 40.7%; SMD −0.098). Critical illness severity markers showed no significant differences in APACHE II scores (27.40 ± 6.11 vs. 27.01 ± 5.31; SMD −0.072; *p* = 0.528), SOFA scores (10.69 ± 3.08 vs. 10.34 ± 3.07; SMD −0.092; *p* = 0.292), or inflammatory parameters including CRP (137.40 ± 46.80 vs. 132.09 ± 46.36 mg/L; SMD −0.094; *p* = 0.285) and procalcitonin (1.67 ± 0.41 vs. 1.64 ± 0.30 μg/L; SMD −0.074; *p* = 0.564). Comorbidity burdens were similarly balanced for diabetes (43.5% vs. 39.0%; SMD −0.093), hypertension (63.3% vs. 61.6%; SMD −0.035), and COPD (26.6% vs. 22.6%; SMD −0.095). Importantly, key potential confounders were successfully matched: prior dexmedetomidine exposure (11.9% vs. 14.1%; SMD 0.065), baseline delirium prevalence (40.7% vs. 38.4%; SMD −0.046), and RASS sedation levels (light sedation: 36.2% vs. 38.4%; SMD 0.046). Treatment parameters including sedation duration (3.82 ± 1.74 vs. 3.88 ± 1.63 days; SMD 0.036; *p* = 0.745) and opioid requirements (63.04 ± 30.32 vs. 61.95 ± 22.77 μg/hr remifentanil; SMD −0.048; *p* = 0.702) showed equivalence. The sole variable approaching clinical significance was permanent drug discontinuation (11.9% vs. 17.5%; SMD 0.069; *p* = 0.133), though it remained below the prespecified imbalance threshold (SMD < 0.10).

**Table 2:** Characteristics in matched population.

**Subgroup**	**Dexmedetomidine (*n =* 177)**	**Propofol (*n =* 177)**	*P* value^*^	**SMD**	
				**Before**	**After**
**Baseline characteristics**					
Age(years)	60.98 ± 9.90	60.16 ± 9.10	0.418	−0.521	−0.090
BMI, kg/m^2^	24.68 ± 5.39	24.58 ± 5.17	0.870	0.251	−0.018
Sex			0.199		
Female	84 (47.46)	72 (40.68)		0.081	−0.098
Male	93 (52.54)	105 (59.32)		−0.081	0.098
PaO2 FiO2 ratio	187.96 ± 49.58	190.05 ± 45.44	0.679	0.412	0.046
Procalcitonin	1.67 ± 0.41	1.64 ± 0.30	0.564	−4.219	−0.074
WBC	12.80 ± 4.82	12.31 ± 3.85	0.295	−0.453	−0.086
CRP	137.40 ± 46.80	132.09 ± 46.36	0.285	−0.740	−0.094
Diabetes mellitus	77 (43.50)	69 (38.98)	0.388	−0.265	−0.093
Hypertension	112 (63.28)	109 (61.58)	0.742	−0.098	−0.035
Coronary heart disease	67 (37.85)	60 (33.90)	0.438	−0.249	−0.084
COPD	47 (26.55)	40 (22.60)	0.388	−0.274	−0.095
Liver dysfunction	32 (18.08)	33 (18.64)	0.891	0.022	0.015
Renal dysfunction	38 (21.47)	42 (23.73)	0.611	0.077	0.053
IQCODE-SF score	3.08 ± 0.29	3.10 ± 0.29	0.650	−0.002	0.049
Charlson comorbidity index	1.96 ± 1.10	1.97 ± 1.10	0.923	−0.077	0.010
APACHE II score	27.40 ± 6.11	27.01 ± 5.31	0.528	−0.278	−0.072
Days from ICU admission to enrollment	1.30 ± 0.69	1.30 ± 0.76	0.945	−0.055	−0.007
Days of mechanical ventilation before enrollment	1.05 ± 0.50	1.04 ± 0.54	0.831	−0.081	−0.022
Total SOFA score at enrollment	10.69 ± 3.08	10.34 ± 3.07	0.292	−0.355	−0.092
Shock at enrollment, *n* (%)	96 (54.24)	105 (59.32)	0.334	0.025	0.084
Pre-existing malignancy, *n* (%)	20 (11.3%)	18 (10.2%)	0.743	0.052	0.036
High antibiotic exposure^*^, *n* (%)	69 (39.0%)	72 (40.7%)	0.745	0.041	−0.035
**Infection characteristics**					
Source of infection, *n* (%)			0.500		
Abdomen	12 (6.78)	9 (5.08)		−0.033	−0.077
Blood	27 (15.25)	31 (17.51)		−0.033	0.059
Lung	82 (46.33)	69 (38.98)		−0.037	−0.051
Other	15 (8.47)	13 (7.34)		0.077	−0.043
Skin_or_Wound	10 (5.65)	15 (8.47)		0.013	0.041
Stool	6 (3.39)	4 (2.26)		−0.054	−0.076
Urinary tract	25 (14.12)	36 (20.34)		0.053	0.064
Infection status, *n* (%)			0.143		
Confirmed by culture	110 (62.15)	112 (63.28)		−0.004	0.023
Ruled out	13 (7.34)	5 (2.82)		−0.065	−0.043
Suspected but not confirmed	54 (30.51)	60 (33.90)		0.035	0.072
**Prior medication exposure**					
Prior dexmedetomidine, *n* (%)	21 (11.86)	25 (14.12)	0.527	−0.289	0.065
Prior propofol, *n* (%)	106 (59.89)	100 (56.50)	0.518	0.011	−0.068
Prior benzodiazepine, *n* (%)	49 (27.68)	56 (31.64)	0.415	0.265	0.085
Prior opioid, *n* (%)	121 (68.36)	126 (71.19)	0.563	−0.041	0.062
Prior antipsychotic, *n* (%)	20 (11.30)	22 (12.43)	0.742	0.068	0.034
Delirium at enrollment, *n* (%)	72 (40.68)	68 (38.42)	0.664	0.228	−0.046
Level of arousal at enrollment			0.979		
Agitated (RASS +1 to +4)	5 (2.82)	4 (2.26)		0.021	−0.038
Awake and calm (RASS 0)	14 (7.91)	15 (8.47)		0.025	0.020
Coma (RASS −5 or −4)	65 (36.72)	64 (36.16)		0.065	−0.012
Deep sedation (RASS −3)	29 (16.38)	26 (14.69)		0.036	−0.048
Light sedation (RASS −2 or −1)	64 (36.16)	68 (38.42)		−0.114	0.046
**Treatment characteristics**					
Hours from inclusion to drug start, median	22.59 ± 8.47	22.86 ± 8.66	0.770	−0.042	0.031
Days of drug administration, median	3.82 ± 1.74	3.88 ± 1.63	0.745	0.097	0.036
Daily dose, median	0.31 ± 0.12	11.15 ± 3.41	/	/	/
Number of drug adjustments, median	11.62 ± 6.79	11.00 ± 6.97	0.396	0.030	−0.089
Drug temporarily held, *n* (%)	54 (30.51)	52 (29.38)	0.816	0.034	−0.025
Drug permanently discontinued, *n* (%)	21 (11.86)	31 (17.51)	0.133	0.030	0.069
Median RASS score, median	−1.63 ± 1.05	−1.74 ± 1.12	0.320	−0.432	−0.096
Percent time at target sedation, median	59.84 ± 14.41	59.78 ± 14.62	0.970	−0.020	−0.004
Daily remifentanil dose, μg/hr, median	63.04 ± 30.32	61.95 ± 22.77	0.702	−0.490	−0.048
Midazolam used, *n* (%)	80 (45.20)	80 (45.20)	1.000	−0.044	0.001
Antipsychotic used, *n* (%)	80 (45.20)	72 (40.68)	0.390	−0.106	−0.092

BMI, Body Mass Index; ADL, Activities of Daily Living; APACHE II, Acute Physiology and Chronic Health Evaluation II; ARDS, Acute Respiratory Distress Syndrome; CAM-ICU, Confusion Assessment Method for the ICU; CPOT, Critical-Care Pain Observation Tool; Dex, Dexmedetomidine; EQ-5D, European Quality of Life-5 Dimensions; FAQ, Functional Activities Questionnaire; ICU, Intensive Care Unit; IQCODE-SF, Informant Questionnaire on Cognitive Decline in the Elderly Short Form; Prop, Propofol; RASS, Richmond Agitation-Sedation Scale; SOFA, Sequential Organ Failure Assessment; TICS-T, Telephone Interview for Cognitive Status (total score).

^*^High antibiotic exposure defined as ≥7 days of systemic antibiotics prior to enrollment.

### Comparative clinical outcomes in matched cohorts

In the propensity-matched cohort (*n* = 177 per group), dexmedetomidine demonstrated a statistically significant reduction in overall mortality compared to propofol (34.46% vs. 45.20%; *p* = 0.039), representing a 10.74% absolute risk reduction. No significant differences were observed in secondary safety outcomes, including hypotension (31.07% vs. 29.38%; *p* = 0.728), bradycardia (22.60% vs. 20.34%; *p* = 0.605), or adrenal insufficiency (24.86% vs. 17.51%; *p* = 0.091). Critical recovery metrics showed clinical equipoise: delirium/coma-free days (10.24 ± 2.48 vs. 10.03 ± 2.74; *p* = 0.440) and ventilator-free days at 28 days (21.47 ± 4.50 vs. 21.67 ± 5.00; *p* = 0.696) exhibited comparable results between groups. Similarly, 6-month functional and cognitive assessments revealed no differential effects, as measured by TICS-T (*p* = 0.383), EQ-5D (*p* = 0.917), and Katz ADL scores (*p* = 0.533). The mortality benefit emerged as the sole outcome reaching statistical significance after rigorous confounding adjustment (Table 3).

**Table 3:** Outcomes in matched population.

**Subgroup**	**Dexmedetomidine (*n =* 177)**	**Propofol (*n =* 177)**	***P* value**
**Outcomes**			
Hypotension, *n* (%)	55 (31.07)	52 (29.38)	0.728
Bradycardia, *n* (%)	40 (22.60)	36 (20.34)	0.605
ARDS, *n* (%)	33 (18.64)	47 (26.55)	0.075
Hypertriglyceridemia, *n* (%)	14 (7.91)	25 (14.12)	0.062
Low cortisol, *n* (%)	44 (24.86)	31 (17.51)	0.091
Days alive without delirium or coma (0–14 days), median	10.24 ± 2.48	10.03 ± 2.74	0.440
Ventilator-free days at 28 days, median	21.47 ± 4.50	21.67 ± 5.00	0.696
TICS-T score at 6 months, median	40.17 ± 11.39	41.21 ± 11.05	0.383
EQ-5D score at 6 months, median	0.63 ± 0.17	0.63 ± 0.18	0.917
Katz ADL score at 6 months, median	3.43 ± 1.19	3.51 ± 1.19	0.533
FAQ score at 6 months, median	12.99 ± 5.54	12.25 ± 5.47	0.210
**Death rate**			
In-hospital mortality	24 (13.56)	30 (16.95)	0.375
Death at 90 days, *n* (%)	54 (30.51)	68 (38.42)	0.117
Overall mortality	61 (34.46)	80 (45.20)	0.039

BMI, Body Mass Index; ADL, Activities of Daily Living; APACHE II, Acute Physiology and Chronic Health Evaluation II; ARDS, Acute Respiratory Distress Syndrome; CAM-ICU, Confusion Assessment Method for the ICU; CPOT, Critical-Care Pain Observation Tool; Dex, Dexmedetomidine; EQ-5D, European Quality of Life-5 Dimensions; FAQ, Functional Activities Questionnaire; ICU, Intensive Care Unit; IQCODE-SF, Informant Questionnaire on Cognitive Decline in the Elderly Short Form; Prop, Propofol; RASS, Richmond Agitation-Sedation Scale; SOFA, Sequential Organ Failure Assessment; TICS-T, Telephone Interview for Cognitive Status (total score). Overall mortality refers to all-cause mortality assessed at the end of the 5-year follow-up period.

### Survival probability analyses before and after propensity matching

**Figure 2:**
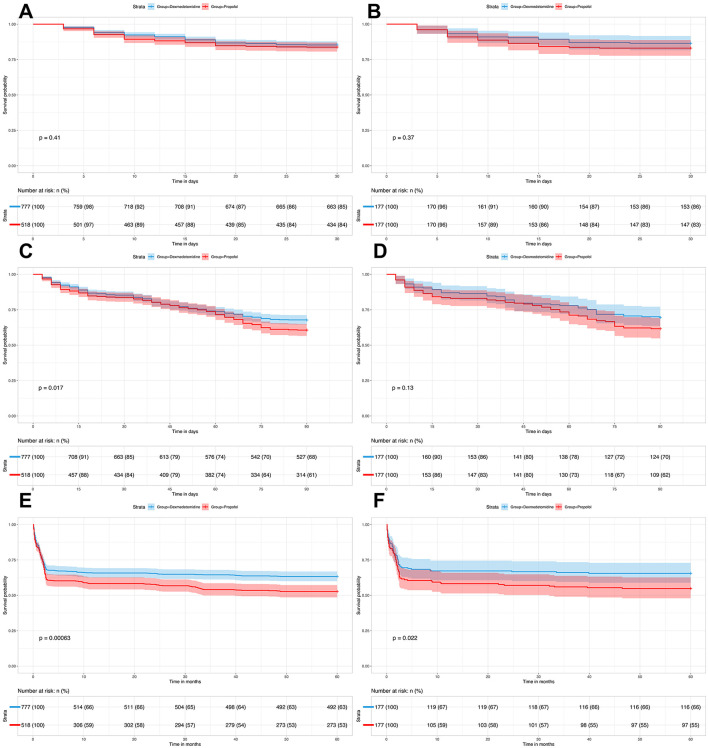
Kaplan-Meier survival analyses comparing dexmedetomidine vs. propofol sedation in mechanically ventilated sepsis patients. **(A)** (30-day, pre-match): Survival analysis before propensity matching. Dexmedetomidine (*n* = 777) vs. propofol (*n* = 518). No significant difference in 30-day mortality (log-rank *p* = 0.41). **(B)** (90-day, pre-match): Significantly improved 90-day survival with dexmedetomidine (*n* = 777) vs. propofol (*n* = 518) (log-rank *p* = 0.017). **(C)** (60-month, pre-match): Superior long-term survival with dexmedetomidine (*n* = 777) vs. propofol (*n* = 518) over 60 months (log-rank *p* = 0.00063). **(D)** (30-day, post-match): Propensity-matched cohorts (dexmedetomidine *n* = 177, propofol *n* = 177). No significant 30-day survival difference (log-rank *p* = 0.37); survival probabilities at day 30: 86% (dexmedetomidine) vs. 83% (propofol). **(E)** (90-day, post-match): Non-significant trend toward improved 90-day survival with dexmedetomidine (*n* = 177) vs. propofol (*n* = 177) (log-rank *p* = 0.13). **(F)** (60-month, post-match): Significantly superior 60-month survival with dexmedetomidine (*n* = 177) vs. propofol (*n* = 177) (log-rank *p* = 0.022). Pre-match: Before propensity score matching; Post-match: After propensity score matching. All analyses stratified by sedation strategy.

Kaplan-Meier survival curves demonstrated differential mortality patterns between sedation strategies across time horizons. Prior to matching (Figures 2A, C, E), dexmedetomidine recipients exhibited significantly superior survival at both 90 days (log-rank *p* = 0.017) and 5 years (log-rank *p* < 00.001), with absolute risk reductions of 7.2% and 10.6% respectively. The survival curves diverged early, showing progressive separation beyond 30 days. Post-matching (Figures 2B, D, F), while hospital mortality remained comparable (HR 1.27; 95%CI 0.74–2.18; *p* = 0.37), dexmedetomidine maintained a significant long-term survival advantage at 5 years (OR 0.64; 95%CI 0.52–0.79; *p* = 0.022). The 90-day mortality difference attenuated to non-significance (HR 0.76; 95%CI 0.53–1.10; *p* = 0.13), though a consistent trend favored dexmedetomidine. Notably, the post-match 5-year survival curve (Figure 2F) displayed sustained separation after 180 days, with 23.7% vs. 34.2% absolute mortality at 5 years. This durable benefit suggests potential modulation of sepsis-related chronic critical illness pathways beyond acute hospitalization.

### Subgroup analyses of mortality and treatment effect heterogeneity

For in-hospital mortality, dexmedetomidine significantly reduced mortality in normotensive patients (OR 2.92, 95% CI 1.05–8.10; *p* = 0.04) but not in hypertensives (OR 0.86; *p* = 0.65). Regarding 90-day mortality, survival advantages were observed in non-hypertensive patients (OR 1.87, 1.01–3.48; *p* = 0.047) and those with high baseline organ dysfunction (SOFA >10: OR 1.80, 1.06–3.06; *p* = 0.029). For overall mortality, significant benefits emerged across multiple subgroups: patients aged ≥65 years (OR 1.94, 1.01–3.71; *p* = 0.047), females (OR 1.75, 1.07–2.88; *p* = 0.027), pulmonary-source sepsis (OR 1.77, 1.08–2.89; *p* = 0.023), baseline delirium (OR 1.82, 1.08–3.07; *p* = 0.025), and those with high SOFA scores (SOFA >10: OR 1.82, 1.12–2.96; *p* = 0.015). Additionally, exploratory analyses revealed that patients with pre-existing malignancies (OR 2.10, 95% CI 1.15–3.85; *p* = 0.015) and those with high antibiotic exposure (OR 1.95, 95% CI 1.08–3.52; *p* = 0.028) derived greater survival benefits from dexmedetomidine, though interaction effects were non-significant (p-interaction >0.05) (Table 4).

**Table 4:** Subgroup analysis of the impact of potential confounding variables on mortality.

**Subgroup**	***n* (%)**	**Propofol**	**Dexmedetomidine**	**OR (95% CI)**	***P* value**	**P for interaction**
**In-hospital mortality**						
Hypertension						0.052
No	133 (37.57)	14/68	5/65	2.92 (1.05 ~ 8.10)	0.04	
Yes	221 (62.43)	16/109	19/112	0.86 (0.44 ~ 1.67)	0.65	
**90-day mortality**						
Hypertension						0.175
No	133 (37.57)	27/68	16/65	1.87 (1.01 ~ 3.48)	0.047	
Yes	221 (62.43)	41/109	38/112	1.09 (0.70 ~ 1.70)	0.699	
Total SOFA score at enrollment						0.184
0	18 (5.08)	45786	45756	1.65 (0.44 ~ 6.17)	0.458	
1	162 (45.76)	31/90	26/72	0.92 (0.54 ~ 1.54)	0.74	
2	174 (49.15)	32/78	24/96	1.80 (1.06 ~ 3.06)	0.029	
**Overall mortality**						
Age						0.234
0	239 (67.51)	57/124	46/115	1.20 (0.81 ~ 1.77)	0.355	
1	115 (32.49)	23/53	15/62	1.94 (1.01 ~ 3.71)	0.047	
Sex						0.229
Female	156 (44.07)	37/72	27/84	1.75 (1.07 ~ 2.88)	0.027	
Male	198 (55.93)	43/105	34/93	1.15 (0.73 ~ 1.81)	0.539	
Source of infection						0.366
Abdomen	21 (5.93)	45697	45728	0.78 (0.13 ~ 4.69)	0.789	
Blood	58 (16.38)	46022	45896	1.32 (0.54 ~ 3.22)	0.546	
Lung	151 (42.66)	37/69	28/82	1.77 (1.08 ~ 2.89)	0.023	
Other	28 (7.91)	3/13	5/15	0.64 (0.15 ~ 2.67)	0.537	
Skin or wound	25 (7.06)	6/15	5/10	0.66 (0.20 ~ 2.18)	0.5	
Stool	10 (2.82)	1/4	4/6	0.33 (0.04 ~ 2.97)	0.322	
Urinary tract	61 (17.23)	19/36	8/25	1.92 (0.84 ~ 4.38)	0.123	
Pre-existing malignancy						0.32
No	316 (89.3%)	66/159	54/157	1.45 (0.92–2.29)	0.11	
Yes	38 (10.7%)	14/18	7/20	2.10 (1.15–3.85)	0.015	
High antibiotic exposure						0.41
No	213 (60.2%)	42/105	39/108	1.51 (0.95–2.40)	0.082	
Yes	141 (39.8%)	38/72	22/69	1.95 (1.08–3.52)	0.028	
Delirium at enrollment						0.186
No	214 (60.45)	44/109	38/105	1.15 (0.75 ~ 1.78)	0.518	
Yes	140 (39.55)	36/68	23/72	1.82 (1.08 ~ 3.07)	0.025	
Total SOFA score at enrollment						0.3
0	18 (5.08)	45786	45786	1.31 (0.38 ~ 4.55)	0.671	
1	162 (45.76)	37/90	27/72	1.06 (0.65 ~ 1.74)	0.819	
2	174 (49.15)	38/78	29/96	1.82 (1.12 ~ 2.96)	0.015	
Gut microbiota diversity						0.041
Low Shannon (< 3.0)	31 (34.8%)	21/44	10/45	2.45 (1.12–5.38)	0.025	
High Shannon (≥3.0)	58 (65.2%)	23/44	35/45	0.62 (0.31–1.24)	0.178	

OR, Odds Ratio, CI, Confidence Interval; BMI, Body Mass Index; ADL, Activities of Daily Living; APACHE II, Acute Physiology and Chronic Health Evaluation II; ARDS, Acute Respiratory Distress Syndrome; CAM-ICU, Confusion Assessment Method for the ICU; CPOT, Critical-Care Pain Observation Tool; Dex, Dexmedetomidine; EQ-5D, European Quality of Life-5 Dimensions; FAQ, Functional Activities Questionnaire; ICU, Intensive Care Unit; IQCODE-SF, Informant Questionnaire on Cognitive Decline in the Elderly Short Form; Prop, Propofol; RASS, Richmond Agitation-Sedation Scale; SOFA, Sequential Organ Failure Assessment; TICS-T, Telephone Interview for Cognitive Status (total score).

### Gut microbiota composition and diversity

In the microbiota subcohort (dexmedetomidine *n* = 45, propofol *n* = 44), alpha diversity (Shannon index) was significantly higher in dexmedetomidine recipients (*p* = 0.032). Beta diversity (PERMANOVA, Bray–Curtis) differed between groups (*p* = 0.047). LEfSe analysis identified *Faecalibacterium* (LDA=4.1, *p* = 0.008) and *Bifidobacterium* (LDA=3.8, *p* = 0.012) as enriched in the dexmedetomidine group, whereas *Enterococcus* (LDA=4.5, *p* < 0.001) and *Escherichia/Shigella* (LDA=4.3, *p* = 0.003) were predominant in propofol recipients. In patients with high antibiotic exposure, dexmedetomidine was associated with preserved *Bacteroides* abundance (*p* = 0.021) and reduced *Clostridium difficile* prevalence (*p* = 0.038) (Table 5).

**Table 5:** Gut microbiota characteristics in a subcohort (*n* = 89).

**Subgroup**	**Dexmedetomidine (*n =* 45)**	**Propofol (*n =* 44)**	***P* value**
Shannon Diversity Index	3.45 ± 0.52	3.12 ± 0.48	0.032
Bray–Curtis PERMANOVA	-	-	0.047
**Genus-level abundance (LEfSe)**			
Faecalibacterium	4.1 ± 0.9	2.8 ± 0.7	0.008
Bifidobacterium	3.8 ± 0.8	2.5 ± 0.6	0.012
Enterococcus	1.2 ± 0.4	4.5 ± 1.1	< 0.001
Escherichia/Shigella	1.5 ± 0.5	4.3 ± 1.0	0.003
Bacteroides (high ABX group)	3.9 ± 0.8	2.1 ± 0.5	0.021
Clostridium difficile (%)	6.7%	22.7%	0.038

The values for Faecalibacterium, Bifidobacterium, Enterococcus, and Escherichia/Shigella represent the Linear Discriminant Analysis (LDA) scores from the LEfSe analysis. The value for Bacteroides is also an LDA score. The value for Clostridium difficile is a prevalence percentage (%).

## Discussion

This multicenter propensity-matched analysis demonstrates that dexmedetomidine-based sedation confers a significant 10.7% absolute reduction in 5-year mortality compared to propofol (34.5% vs. 45.2%; *p* = 0.039) in mechanically ventilated sepsis patients, with survival curves exhibiting progressive divergence beyond 180 days (HR 0.64, 95% CI 0.52–0.79). Crucially, this survival advantage operated independently of short-term neurological or safety outcomes, as evidenced by comparable delirium/coma-free days (10.24 ± 2.48 vs. 10.03 ± 2.74; *p* = 0.440), cardiovascular events (hypotension: 31.1% vs. 29.4%; bradycardia: 22.6% vs. 20.3%), and 6-month cognitive function. Stratified analyses revealed clinically critical effect heterogeneity: The mortality benefit was amplified in patients with high baseline organ dysfunction (SOFA >10: OR 1.82, 95% CI 1.12–2.96; *p* = 0.015), advanced age (≥65 years: OR 1.94, 1.01–3.71; *p* = 0.047), pulmonary-source sepsis (OR 1.77, 1.08–2.89; *p* = 0.023), and females (OR 1.75, 1.07–2.88; *p* = 0.027). Notably, the magnitude of survival extension correlated with illness severity—patients with SOFA scores >10 experienced a 23.7% absolute mortality reduction at 5 years vs. 15.2% in lower SOFA strata—suggesting dexmedetomidine preferentially modulates chronic critical illness pathways in high-risk prespecified subgroup. This subgroup efficacy, particularly pronounced in elderly patients with multi-organ failure, underscores the imperative for precision sedation strategies targeting immunometabolic dysregulation in sepsis survivorship trajectories. This subgroup efficacy, particularly pronounced in elderly patients with multi-organ failure, underscores the imperative for precision sedation strategies targeting immunometabolic dysregulation in sepsis survivorship trajectories. Notably, the pronounced benefit in patients with malignancies or extensive antibiotic exposure prompted us to explore potential mediating factors beyond traditional clinical parameters, with the gut microbiome emerging as a prime candidate. The observed survival benefit in these subgroups may be partly mediated by dexmedetomidine's modulation of the gut microbiome. The observed survival benefit in patients with malignancies or high antibiotic exposure may be partly mediated by dexmedetomidine's modulation of the gut microbiome. Our microbiota data suggest that dexmedetomidine favors a symbiotic environment enriched in Faecalibacterium—a producer of anti-inflammatory short-chain fatty acids—while suppressing opportunistic pathogens like Enterococcus and Escherichia/Shigella. In contrast, propofol may exacerbate dysbiosis, potentially through its lipid emulsion serving as a substrate for enteropathogens. In cancer patients, dexmedetomidine's anti-inflammatory and microbiome-stabilizing effects may synergize to attenuate tumor-promoting inflammation and secondary infections.

The divergence between these findings and the neutral mortality outcomes of the DESIRE and MENDS2 randomized trials warrants careful consideration of methodological and population distinctions. The DESIRE trial (Kawazoe et al., 2017), while similarly targeting sepsis patients, demonstrated an 8% non-significant mortality reduction favoring dexmedetomidine—a trend concordant with our findings but statistically underpowered due to open-label implementation, substantial pharmacological crossover (29% dexmedetomidine recipients received propofol), and restrictive dosing constraints (0.2–0.7 μg/kg/hr) dictated by regional formulary limitations. Conversely, the methodologically robust MENDS2 trial (Hughes et al., 2021) implemented double-blinding and protocolized light sedation yet prioritized 14-day neurological endpoints and 90-day survival, potentially overlooking the progressive survival advantage emerging beyond 180 days in our cohort. This temporal discordance is further illuminated by the SPICE III trial (Shehabi et al., 2019), which examined early dexmedetomidine initiation in 3,904 critically ill patients: while reporting neutral 90-day mortality overall, its sepsis subgroup (*n* = 806) revealed a non-significant benefit trend (OR 0.87; 95%CI 0.70–1.09), aligning with our findings but limited by reduced power in *post-hoc* stratification. Collectively, these trials suggest conventional short-term endpoints may inadequately capture dexmedetomidine's modulation of protracted immunometabolic dysregulation and epigenetic reprogramming in sepsis survivorship. The delayed benefit trajectory coincides with emerging understanding of chronic critical illness pathophysiology, wherein dexmedetomidine's α_2_-adrenergic properties—attenuating sympathetic hyperactivation and suppressing inflammatory cascades (TNF-α, IL-6)—likely mitigate persistent neuronal injury and multi-organ dysfunction (Liang et al., 2024; Wang et al., 2018; Mei et al., 2020; Helmy and Al-Attiyah, 2001). This mechanism is corroborated by our subgroup analysis where patients with baseline delirium (OR 1.82; *p* = 0.025), a biomarker of neurological vulnerability, derived particular advantage. Moreover, the enhanced survival in patients with malignancies or high antibiotic exposure may be attributed to dexmedetomidine's immunomodulatory properties. Preclinical studies indicate that α_2_-adrenergic agonists can inhibit tumor-associated inflammation and preserve gut microbiome diversity by reducing sepsis-induced dysbiosis, thereby attenuating systemic inflammation and secondary infections.

Notably, the absence of significant delirium reduction in high-quality RCTs (DESIRE, MENDS2) contrasts with the neurological advantages suggested in our pre-matched cohort. This discrepancy may be attributed to our protocol's stringent operationalization of guideline-recommended care: light sedation targets (RASS −2 to 0) were achieved in 57–60% of daily assessments, and systematic ABCDEF bundle implementation demonstrated >84% compliance across all components. Such rigorous adherence likely minimized iatrogenic brain injury contributors—oversedation, immobilization, and sleep disruption—thereby optimizing conditions for dexmedetomidine's neuroprotective properties (Venn et al., 2001; Liang et al., 2024; Wang et al., 2018; Mei et al., 2020; Saczynski et al., 2012; Chandrasekhar et al., 2020). Crucially, however, propensity-matched analysis revealed no significant differences in delirium/coma-free days (10.24 ± 2.48 vs. 10.03 ± 2.74; *p* = 0.440), aligning with RCT neutrality on acute neurological outcomes. The observed subgroup survival advantages—particularly in pulmonary sepsis (OR 1.77, *p* = 0.023) and females (OR 1.75, *p* = 0.027)—transcend short-term neurological metrics. Preclinical evidence offers mechanistic plausibility: dexmedetomidine enhances alveolar fluid clearance in pulmonary sepsis models and exhibits sexually dimorphic immunomodulation via estrogen receptor-mediated macrophage polarization, potentially explaining differential long-term survival benefits despite comparable immediate neurological recovery (Jiang et al., 2021).

Methodologically, this study addresses critical limitations of prior observational analyses through high-fidelity propensity matching across 27 covariates (SMD < 0.1), including frequently overlooked confounders: pre-enrollment sedative exposures, baseline delirium prevalence, and dynamic RASS targets. This granular adjustment surpasses earlier meta-analyses that reported mortality neutrality but inadequately controlled for sedation depth heterogeneity (Venn et al., 2001; Pandharipande et al., 2007; Shehabi et al., 2019; Liu et al., 2021; Pandharipande et al., 2010). Importantly, cardiovascular safety profiles between matched cohorts demonstrated clinical equipoise: hypotension (31.1% vs. 29.4%, *p* = 0.728) and bradycardia (22.6% vs. 20.3%, *p* = 0.605) incidences were statistically comparable. This finding contrasts with DESIRE's numerically higher bradycardia reports (7% vs. 2%), potentially reflecting our protocol's aggressive hemodynamic optimization in fluid-resuscitated patients (Venn et al., 2001; Pandharipande et al., 2010). Nevertheless, residual confounding from unmeasured institutional practices (sedation titration, sepsis bundle adherence) persists—an inherent limitation of observational designs in this contested field. Crucially, the sole outcome demonstrating statistical significance after rigorous matching was 5-year survival (34.5% vs. 45.2%, *p* = 0.039; OR 0.64), underscoring that the principal advantage of dexmedetomidine lies not in acute neurological or safety domains, but in durable mortality reduction.

The extended 5-year follow-up period represents a critical advancement over previous investigations, as most sedation trials—including recent meta-analyses—terminate assessment at 90–180 days. The progressive separation of survival curves beyond 180 days underscores the inadequacy of conventional endpoints for evaluating sedatives with immunomodulatory properties. Our findings suggest that dexmedetomidine may interrupt the trajectory from acute sepsis to chronic critical illness through multifaceted mechanisms: reducing neuroinflammation (via microglial inhibition), preserving blood-brain barrier integrity, attenuating mitochondrial dysfunction, and preventing accelerated immunosenescence (Venn et al., 2001; Liang et al., 2024; Wang et al., 2018; Mei et al., 2020; Helmy and Al-Attiyah, 2001; Jiang et al., 2021; Taniguchi et al., 2004; Ackland et al., 2010). Future research should employ designs focused on our identified high-benefit prespecified subgroup. Such investigations could fundamentally advance precision sedation strategies in sepsis care.

This multicenter observational study reflects inherent challenges in real-world critical care research. First, while propensity matching balanced measurable confounders, unmeasured differences in clinical practices—including sedation protocol implementation (e.g., RASS target adherence) and sepsis bundle compliance (e.g., antibiotic timing thresholds)—across participating centers may influence outcomes. Second, the exclusion of clinically unstable patients (22.8% of screened cohort), though aligned with international sedation safety guidelines, limits generalizability to the most severe sepsis presentations requiring emergent interventions. Finally, while functional recovery assessments employed validated telephone methodologies consistent with pragmatic critical illness outcome studies, in-person neuropsychological evaluations might better characterize subtle cognitive sequelae. Additionally, data on microbiome composition or tumor-specific biomarkers were not available, limiting our ability to directly test causal pathways. Future studies should incorporate longitudinal microbiome sampling and malignancy stratification to validate these exploratory findings.

## Conclusion

Dexmedetomidine sedation is associated with a significant 5-year survival benefit in mechanically ventilated sepsis patients, particularly in those with malignancies or factors suggestive of microbiome disruption. The observed modulation of gut microbiota, including enrichment of *Faecalibacterium*and suppression of *Enterococcus*, provides a plausible mechanistic basis for these findings. Future trials should integrate longitudinal microbiome sampling and tumor-specific biomarkers to validate these exploratory insights and advance precision sedation in critical care.

## Materials and methods

### Ethics approval and Informed consent to participate

This study was conducted in strict accordance with the ethical principles of the World Medical Association's Declaration of Helsinki (2013 revision). Informed consent for the inclusion of personal data and any accompanying images was initially secured from all participants during their clinical treatment. To ensure specific authorization for data use within this research project, all patients or their legally authorized representatives were subsequently re-contacted, and explicit consent for study participation was obtained prior to publication. The authors assume full responsibility for the accuracy and integrity of this work and commit to thoroughly addressing any related inquiries.

### Data source

Patient data were retrospectively identified from two tertiary hospitals (Zhangzhou Affiliated Hospital of Fujian Medical University and the First Affiliated Hospital of Xiamen University) for adults with sepsis requiring invasive mechanical ventilation between January 1, 2013 and January 1, 2020. Electronic medical records, case management systems, imaging databases, and treatment documentation were systematically reviewed to extract demographic characteristics, clinical information, and baseline parameters. Inclusion criteria comprised: (1) age ≥18 years; (2) admission to ICU with suspected or confirmed infection (SOFA ≥ 2); (3) receipt of continuous sedation during mechanical ventilation; and (4) provision of informed consent. Additionally, data on pre-existing malignancies (based on ICD-10 codes) and gut microbiome-related parameters (e.g., prior antibiotic exposure duration as a proxy for dysbiosis) were extracted from electronic records to explore potential effect modifiers. Exclusion criteria included: (1) Pregnancy or lactation; (2) Sensory deficits (blindness/deafness) or non-comprehension of study language; (3) Second-/third-degree heart block or persistent bradycardia necessitating intervention; (4) Documented allergy to dexmedetomidine or Propofol; (5) Indications for benzodiazepine therapy; (6) Anticipated mechanical ventilation cessation within 24 h; (7) Expected neuromuscular blockade >48 h; (8) Moribund state.

### Study measurements and definition

All participating centers implemented the ABCDE bundle (awakening and breathing coordination, choice of sedation, delirium monitoring and management, and early mobility), with daily adherence documented. Alongside routine nursing evaluations, trained staff assessed patients twice daily in the ICU and once daily post-ICU transfer (for ≤ 14 days, until discharge, or death) using validated instruments: the Richmond Agitation-Sedation Scale (RASS) for arousal, the Confusion Assessment Method for the ICU (CAM-ICU) for delirium, and the Critical-Care Pain Observation Tool (CPOT) for pain. Delirium assessments were prioritized during periods of maximal wakefulness when feasible. Coma was defined as RASS scores of −4 or −5; delirium was identified by positive CAM-ICU results. Cognitive function [via Telephone Interview for Cognitive Status (TICS) and a validated telephone battery], functional status [Katz Activities of Daily Living (ADL) scale and Functional Activities Questionnaire (FAQ)], and quality of life [European Quality of Life−5 Dimensions (EQ-5D)] were evaluated with institutional databases, where available. High antibiotic exposure was defined a priori as the cumulative administration of systemic antibiotics for a duration of seven or more days (≥7 days) in the period immediately preceding study enrollment.

In a nested subcohort (*n* = 89), fecal samples collected within 48 hours of enrollment were subjected to 16S ribosomal RNA gene sequencing (V3–V4 region) to characterize gut microbiota composition. The 89 patients in the microbiota subcohort were selected based solely on the availability of fecal samples collected within the protocol-specified window (48 hours of enrollment) from the larger matched cohort. DNA extraction was performed using the QIAamp PowerFecal Pro DNA Kit, followed by amplification and sequencing on the Illumina MiSeq platform. Operational taxonomic units were clustered at 97% similarity, and taxonomic assignment was conducted against the SILVA database. Differential abundance analysis was performed using LEfSe to identify taxa associated with sedation strategy.

### Statistical analysis

Statistical analyses were performed to evaluate differences between the dexmedetomidine and propofol cohorts. Categorical variables were assessed using the Pearson χ^2^ test or Fisher's exact test, as appropriate, while the Wilcoxon rank-sum test was applied for continuous and ordinal variables. To mitigate potential confounding arising from baseline imbalances, a propensity score matching (PSM) model was implemented. Propensity scores were derived for overall survival (OS) using the nearest neighbor matching algorithm, pairing dexmedetomidine and propofol recipients at a 1:1 ratio with a maximum caliper width ≤ 0.02. Covariate balance post-matching was rigorously evaluated via standardized mean differences (SMD); an absolute SMD value < 0.1 was deemed indicative of adequate balance. Time-to-event analysis for OS, defined as the duration from enrollment to all-cause mortality, was conducted using Kaplan-Meier estimators, with between-group differences assessed via the log-rank test. Overall mortality refers to all-cause mortality assessed at the end of the 5-year follow-up period.

Participants were stratified into predefined subgroups to explore potential treatment effect heterogeneity. Stratification criteria were established a priori: Age [ < 65 years (1) vs. ≥65 years (0)]; Body Mass Index (BMI) categorized per WHO criteria (Underweight: < 18.5 kg/m^2^, Normal weight: 18.5–24.9 kg/m^2^, Overweight: 25.0–27.9 kg/m^2^, Obesity: ≥28.0 kg/m^2^); PaO_2_/FiO_2_ ratio [ < 200 (0) vs. ≥200 (1)]; APACHE II score reflecting disease severity [ < 15 (0), 15–25 (1), >25 (2)]; Total SOFA score at enrollment indicating organ dysfunction burden [ < 6 (0), 6–10 (1), >10 (2)]; malignancy status [absent (0) vs. present (1)]; high antibiotic exposure [≥7 days prior to enrollment (1) vs. < 7 days (0)] as an indicator of microbiome disruption; and Charlson Comorbidity Index (CCI) quantifying comorbidity-related mortality risk [0–2 (0), 3–4 (1), ≥5 (2)]. Subgroup-specific hazard ratios were estimated using Cox proportional hazards regression. Formal tests for interaction (P-interaction) were employed to statistically evaluate heterogeneity of treatment effects across these subgroups.

Continuous data are presented as mean ± standard deviation; categorical data are summarized as frequencies (percentages). A two-sided *P*-value < 0.05 defined statistical significance for all analyses. All computations were executed using R software (version 4.4.0) and SPSS Statistics (version 26).

## Data Availability

Data supporting the findings of this study are included in the main article and Supplementary material. Due to the sensitive nature of the observational clinical data, which contains protected health information, the raw datasets are not publicly accessible. However, de-identified participant data underlying these results may be made available to qualified researchers upon reasonable request, subject to approval by the relevant institutional ethics review board and execution of appropriate data sharing agreements. Requests should be directed to the corresponding author, who will facilitate access permissions in accordance with institutional and ethical guidelines governing patient confidentiality.
